# Prevalence of the Human Immunodeficiency Virus and associated factors in pregnant women in the state of Pará

**DOI:** 10.1590/0034-7167-2021-0171

**Published:** 2022-06-24

**Authors:** Helloyza Halana Fernanda Aquino Pompeu, Lilia Pimenta de Moraes, Camila Cristina Girard Santos, Helber Yanaguibashi Shibata, Jonas Elias Castro da Rocha, Alexandre Aguiar Pereira, Conceição do Socorro Damasceno Barros, Cristiane Patrícia Siqueira Monteiro

**Affiliations:** I Universidade Federal do Pará. Belém, Pará, Brazil.; II Universidade do Estado do Pará. Belém, Pará, Brazil.; III Universidade Federal Rural da Amazônia. Belém, Pará, Brazil.; IV Centro Universitário do Estado Pará. Belém, Pará, Brazil.

**Keywords:** Pregnant Women, HIV Seropositivity, Prevalence, Attention to Health, Nursing, Mujeres Embarazadas, Seropositividad para VIH, Prevalencia, Atención a la Salud, Enfermería, Gestantes, Soropositividade para HIV, Prevalência, Atenção à Saúde, Enfermagem

## Abstract

**Objectives:**

to analyze the prevalence of the Human Immunodeficiency Virus and the associated factors in pregnant women in the state of Pará.

**Methods:**

retrospective, analytical, quantitative study with a sample of 332 medical records of HIV-positive pregnant women hospitalized at the Referral Maternity Hospital in the state of Pará between 2010 and 2019. Bivariate and multivariate statistical analysis were performed with the variables collected.

**Results:**

the average prevalence in the period was 2.39% and the Metropolitan Region concentrated 66.87% of cases. There was a strong relationship between the number of antenatal consultations and lack of knowledge of serological status (p value equal to 0.01^E-17^) variables, and a correlation between the education and number of antenatal consultations variables.

**Conclusions:**

the increase in the infection rate during the study period revealed the need to intensify health actions, early diagnosis and strategies to improve adherence to antiretroviral treatment for maternal viral suppression and reduction of the risk of vertical transmission, contributing to improve public policies.

## RESUMO

**Objetivos:**

analisar a prevalência do Vírus da Imunodeficiência Humana e os fatores associados em gestantes no estado do Pará.

**Métodos:**

estudo analítico, quantitativo e retrospectivo com a amostra de 332 prontuários de gestantes HIV positivas internadas na Maternidade de Referência do estado do Pará, no período de 2010 a 2019. Com as variáveis coletadas, procedeu-se a análise estatística bivariada e multivariada.

**Resultados:**

a média de prevalência no período foi de 2,39% e a Região Metropolitana concentrou 66,87% dos casos. Houve forte relação entre as variáveis número de consultas pré-natais e desconhecimento do *status* sorológico (*p* valor igual a 0,01^E-17^) e correlação entre as variáveis escolaridade com o número de consultas pré-natais.

**Conclusões:**

o aumento da taxa de infecção no período estudado revelou a necessidade de intensificar as ações de saúde, o diagnóstico precoce e as estratégias para a melhoria da adesão ao tratamento antirretroviral para supressão viral materna e redução do risco de transmissão vertical, contribuindo para aprimorar as políticas públicas.

## RESUMEN

**Objetivos:**

analizar la prevalencia del Virus de la Inmunodeficiencia Humana y los factores asociados en mujeres embarazadas en el estado de Pará.

**Métodos:**

estudio retrospectivo, analítico, cuantitativo con una muestra de 332 historias clínicas de gestantes VIH positivas hospitalizadas en la Maternidad de Referencia del estado de Pará entre 2010 y 2019. Se realizó análisis estadístico bivariado y multivariado con las variables recolectadas.

**Resultados:**

la prevalencia promedio en el período fue de 2,39% y la Región Metropolitana concentró el 66,87% de los casos. Hubo fuerte relación entre las variables número de consultas prenatales y desconocimiento del estado serológico (valor de p igual a 0,01^E-17^) y correlación entre las variables educación y número de consultas prenatales.

**Conclusiones:**

el aumento de la tasa de infección durante el período de estudio reveló la necesidad de intensificar las acciones de salud, el diagnóstico precoz y las estrategias para mejorar la adherencia al tratamiento antirretroviral para la supresión viral materna y la reducción del riesgo de transmisión vertical, contribuyendo a mejorar las políticas públicas.

## INTRODUCTION

Human Immunodeficiency Virus (HIV) infection is a serious public health problem in the world that seriously threatens the health and lives of women and children. In 2018, 37.9 million people were living with HIV worldwide, of which 49.6% consisted of women over 15 years of age and 4.5% were children under 15 years^(1)^. In this context, the infection affects mainly women of reproductive age, causing a greater risk of vertical transmission (VT)^(2)^.

The main means of transmission to children are HIV infection during pregnancy, childbirth and breastfeeding. In Brazil, it is estimated that approximately 0.38% of pregnant women are infected with HIV, which corresponds to about 11,000 women per year. Antenatal and childbirth coverage in the country is close to 100%, and medications and breastmilk substitutes for the prevention of VT of HIV have been in use since the 1990s^(3)^. When used in a timely and appropriate manner, these prophylactic measures can reduce transmission to less than 2%. Without any intervention, the overall transmission rate is between 15% and 45%^(4)^.

In 2019, 8,312 HIV-infected pregnant women were identified in Brazil, 12.5% of which in the North region. Note that 12 Federation Units (FU) had HIV detection rates in pregnant women higher than the national rate, among which the state of Pará that reached 3.7/thousand live births, placing the state in fifth position. When analyzing the detection rates of the Acquired Immunodeficiency Syndrome (AIDS) between capitals, the highest was found in Belém (8.3/100 thousand inhabitants)^(5)^. The main public Maternity Hospital in the state, located in the capital Belém, is the largest maternal and child referral center in the North region and consequently receives most cases of pregnant women living with HIV. The last study on the prevalence of HIV in this population was conducted in 2011, covering the period from 2004 to 2010, and a prevalence rate of 1.87% was identified in the Institution^(6)^.

HIV testing of pregnant women and their partners, as well as condom use and family planning have been the main strategies implemented to achieve infection control. Knowledge of HIV serological status through tests accompanied by counseling can lead to the adoption of less risky behaviors, while the antiretroviral therapy (ART) leads to viral suppression, reducing heterosexual transmission and VT^(7)^.

High burdens of HIV infection in pregnant women can be a barrier to achieve the elimination of new pediatric infections. Thus, it is imperative to understand the clinical, social and geographic factors associated with this type of infection, allowing that HIV control programs focus evidence-based prevention interventions primarily on high-risk women living in more vulnerable geographic areas. Understanding these risk factors and implementing such interventions can also help in the control and/or elimination of pediatric HIV^(7)^.

In view of the above, the study is justified by the fact that the northern region of Brazil and the city of Belém stand out in the national scenario, showing significant growth in the coefficients of HIV detection in pregnant women in the last ten years^(5)^. HIV infection is known to still be very prevalent in more vulnerable regions and drastically affect pregnant women, especially those with unsuppressed viral load or unknown serological status, as they require special care and must have adequate planning of labor to prevent perinatal transmission of the virus to the baby^(8)^.

Therefore, this group requires specialized and targeted care, especially from nursing, both because of the complexity involved in caring for these pregnant women and the differentiated care demanded by the mother-child binomial from antenatal care, a scenario where nurses play the main role^(9)^. It is essential to know the scenario and epidemiological situation of HIV in pregnant women in order to evaluate the possibility of eliminating VT of the virus, identify the associated factors and suggest strategies to combat infection and improve antenatal care, delivery and postpartum^(10)^.

In addition, conducting studies that investigate the prevalence of HIV and associated factors in pregnant women in the state of Pará may allow the visualization of the infection panorama. Differences in detection rates are observed in several regions of the country, mainly in the north and northeast regions, where greater increases in the rate have been found; 83.3% in the last ten years in both^(5)^.

## OBJECTIVES

To analyze the prevalence of the Human Immunodeficiency Virus and the associated factors in pregnant women in the state of Pará.

## METHODS

### Ethical aspects

This study followed the recommendations proposed by the National Health Council in Resolution No. 466/2012, was authorized by the institution and approved by the Research Ethics Committee of a referral public Maternity Hospital in the north region of Brazil. To obtain secondary data, the form of Commitment for the Use of Data was signed to express agreement with the commitment to use and preserve the records handled.

### Design, study location and period

This is a retrospective, analytical, quantitative study guided by the STROBE statement. It was conducted in a high complexity public Maternity Hospital located in the city of Belém (state of Pará) that is the largest maternal and child referral center in the north of Brazil. It has 110 maternity beds and a walk-in Urgency and Emergency Department that serves referrals from the 144 municipalities of the state, providing user embracement, risk classification and testing for HIV, syphilis and hepatitis B and C for hospitalized pregnant women. The study was conducted from August to October 2020.

### Sample, inclusion and exclusion criteria

The study sample consisted of 332 medical records of pregnant women living with HIV hospitalized at the Institution from 2010 to 2019, selected for convenience and respecting the proportionality of hospitalizations per year. The total number of medical records evaluated per year was the following: 26 from 2010; 19 from 2011; 27 from 2012; 29 from 2013; 35 from 2014; 49 from 2015; 41 from 2016; 40 from 2016; 40 from 2017; 29 from 2018; and 37 from 2019. The formula for limited (finite) populations described by Fontelles^(11)^ was used for sample size calculation, based on the number of 1,962 pregnant women living with HIV hospitalized during the study period, according to data from the statistics department of the Institution. A tolerable relative error of 5% and a 95% confidence interval were considered.

The following inclusion criteria were used: medical records of pregnant women living with HIV hospitalized at the Institution between 2010 and 2019 for childbirth and/or clinical treatment. This period was selected because the last study on the prevalence of HIV in pregnant women at the Institution investigated the period from 2004 to 2010. Records without sufficient information for data collection were excluded, that is, in which two or more obstetric, demographic and/or clinical information (variables of interest to the study) were lacking. As 3.05% of medical records were incomplete, those with insufficient information were replaced by others to avoid compromising the sample size.

### Study protocol

Data collection was performed in three steps: 1) presentation of the study to the direction of the department responsible for the archive to facilitate the main researcher’s access to the location and separation of records of interest, according to the Institution’s rules; 2) requesting data from the statistics department of the Institution regarding the number of deliveries performed, number of live births, HIV tests performed and positive tests in the studied period; and 3) application of a structured form to collect data regarding sociodemographic, obstetric and clinical characteristics related to HIV identified in the medical records.

Data collected were organized as epidemiological variables divided into three main axes: 1) Sociodemographic (age, race, marital status, profession/occupation and city of origin); 2) Obstetric (number of pregnancies, deliveries and abortions, live children and antenatal consultations); and 3) HIV-related clinical (knowledge of serological status and follow-up/treatment of HIV during pregnancy). As soon as variables of interest were collected, started the database construction in a software.

### Analysis of results and statistics

The information collected was included in a Microsoft Excel database and analyzed in the Statistical Package for the Social Sciences (SPSS), version 20. Initially, the simple and percentage frequencies of variables were calculated and subsequently, bivariate and multivariate statistics were used.

Pearson’s chi-square test was performed in bivariate analysis, considering a statistical significance level of 5% and a confidence interval of 95% in all tests. For multivariate analysis, the Statistica software, version 9.0, was used, which allowed the principal component analysis (PCA), a mathematical procedure that uses an orthogonal transformation (orthogonalization of vectors) to convert a set of observations of possibly uncorrelated variables into a set of values of linearly correlated variables, called principal components.

Finally, the geographical distribution of pregnant women was performed according to the city of origin using the QGIS program, version 2.18. For this purpose, shapefiles of the municipalities in the state of Pará were acquired from the database of the Brazilian Institute of Geography and Statistics (IBGE). Shapefiles are vector files used in Geographic Information Systems (GIS) that store data about the position, shape and geographic features, simulating the real shape of the Earth and its regions. Thus, the codes, coordinates, names of each municipality and number of pregnant women living with HIV were tabulated in Comma Separated Value (.csv) format and later imported into the QGIS program environment. Finally, data were transformed into files in shapefile format (.shp), according to the Geocentric Reference System for the Americas, also known as SIRGAS 2000, allowing application on the map and classification of the number of cases according to the municipalities of residence of pregnant women.

To calculate HIV prevalence, the number of HIV cases detected in hospitalized pregnant women was divided by the total number of live births at the Maternity Hospital during the study period, using information provided by the statistics department of the Institution. The processed and analyzed data were organized in tables and figures for a clear presentation and dissemination of information regarding the proposed objective.

## RESULTS

A total of 332 medical records of pregnant women living with HIV hospitalized between 2010 and 2019 were evaluated. With regard to sociodemographic characteristics, the 21-30 years age group was the most predominant (58.13%). The mean age was 25.42 years; the lowest age was 13 years old and the highest was 48 years old. Mixed race predominated (94.28%) among pregnant women. As for marital status, pregnant women in a common-law marriage predominated (48.80%). The predominant educational level was complete secondary school (31.33%) and the type of occupation “housewife” was the most recurrent (71.08%).

With regard to obstetric characteristics, pregnant women had at least one antenatal consultation (85.84%) and the average number of consultations was 5.86. Note that a significant number (46.08%) did not perform the ideal number of six antenatal consultations. When evaluating the number of pregnancies, most (76.20%) were multiparous. As for parity, multiparous women predominated (71.93%) and most (72.59%) had not had an abortion.

Regarding HIV-related clinical characteristics, the majority (46.99%) discovered the infection in the current pregnancy, with an important percentage (15.66%) unaware of their serological status at the time of hospitalization. A large part (25.90 %) did not undergo appropriate treatment. Table 1 presents the bivariate analysis of the researched variables related to the prevalence of HIV infection in pregnant women hospitalized in the study period.

**Table 1:** Bivariate analysis of sociodemographic, obstetric and clinical characteristics related to the prevalence of Human Immunodeficiency Virus infection in pregnant women hospitalized from 2010 to 2019, Belém, Pará, Brazil, 2020

Variables	%	*p* value*	Standard deviation
Age			
10 -| 20 years	21.39%	0.75	6.01
21 -| 30 years	58.13%		
31 -| 40 years	18.98%		
41 -| 50 years	1.51%		
Race			
Asian	0.60%	0.98	0.42
White	2.71%		
Black	1.51%		
Mixed race	94.28%		
Not informed	0.90%		
Occupation			
Housewife	71.08%	0.7	0.78
Student	6.33%		
Worker	17.77%		
Not informed	4.82%		
Schooling			
Incomplete primary school	26.81%	0.13	1.31
Complete primary school	26.51%		
Incomplete secondary school	7.83%		
Complete secondary school	31.33%		
Incomplete higher education	1.51%		
Complete higher education	1.20%		
Not informed	4.82%		
Marital status			
Married	7.23%	0.29	0.68
Common-law marriage	48.80%		
Divorced	0.60%		
Widow	0.60%		
Single	40.06%		
Not informed	2.71%		
Pregnancies			
No previous pregnancy	23.49%	0.5	1.55
Previous pregnancy	76.20%		
Not informed	0.30%		
Residence			
Metropolitan region	66.87%	0.53	0.47
Inlands	33.13%		
Births			
Primiparous	28.31%	0.32	1.34
Previous delivery	71.93%		
Not informed	0.30%		
Abortion			
0 (none)	72.59%	0.94	0.62
1 (one)	22.89%		
2 (two)	3.61%		
4 (four)	0.30%		
5 (five)	0.30%		
Not informed	0.30%		
Antenatal consultations			
≥ 6 consultations	50.60%	0.0002	3.64
<6 consultations	46.08%		
Not informed	3.31%		
Antenatal care			
Received	85.84%	**0.01 ^E-17^**	**0.31**
Did not receive	10.84%		
Not informed	3.31%		
Treatment time			
Did not undergo	25.90%	-1234	1.92
< 1 month	4.22%		
< 3 months	9.64%		
< 6 months	28.01%		
< 1 year	1.81%		
> 1 year	29.22%		
Not informed	1.20%		
When did you discover infection			
Current pregnancy	46.99%	0.68	0.93
Previous pregnancy	21.99%		
Unaware	15.66%		
Outside the gestational period	13.55%		
Congenital	0.90%		
Not informed	0.90%		

*p value – Significance level of Pearson’s chi-square test.

The analysis showed the existence of a greater correlation between antenatal care and knowledge of HIV serological status variables with p-value equal to 0.01^E-17^. The results also showed the average prevalence of HIV infection of 2.39% in pregnant women hospitalized in the study period; the lowest prevalence rate was 2.03% in 2011, and the highest was 2.83% in 2019. It was found that 20.69% of pregnant women living with HIV were diagnosed in the Urgency and Emergency Department during their admission to the Maternity Hospital, that is, they were unaware of their serological status and consequently, did not undergo ART, increasing the risk of VT.

In addition, the Maternity Hospital performed 81,943 deliveries (average of 8,194.3 per year) and 57,441 HIV tests (average of 5,744.1 per year) in the period, of which 1,962 tests were positive (average of 196.2 positive tests per year), and 397 of these were detected in the Urgency and Emergency Department (average of 39.7 per year), as shown in Table 2.

**Table 2:** Distribution of the number of deliveries, Human Immunodeficiency Virus tests performed, positive tests identified in the Obstetric Urgency and Emergency Department and the prevalence of Human Immunodeficiency Virus infection in pregnant women hospitalized from 2010 to 2019, Belém, Pará, Brazil, 2020

Year	Number of deliveries	Tests performed	Positive tests	Detected at the OUE*	% of positive tests detected	Prevalence	Incidence
2010	5,475	4,055	149	42	28.19%	2.72%	0.77%
2011	5,906	3,820	120	19	15.83%	2.03%	0.32%
2012	7,019	4,963	171	28	16.37%	2.44%	0.40%
2013	6,600	5,240	144	38	26.39%	2.18%	0.58%
2014	7,708	5,080	184	39	21.20%	2.39%	0.51%
2015	9,433	5,880	206	58	28.16%	2.18%	0.61%
2016	9,976	6,470	225	50	22.22%	2.26%	0.50%
2017	10,326	6,498	238	39	16.39%	2.30%	0.38%
2018	9,550	7,805	243	42	17.28%	2.54%	0.44%
2019	9,950	7,630	282	42	14.89%	2.83%	0.42%
Total	81,943	57,441	1,962	397	20.23%	2.39%	0.48%
Média	8,194.3	5,744.1	196.2	39.7	20.69%	2.39%	0.49%

*OUE – Obstetric Urgency and Emergency.

**Figure 1:**
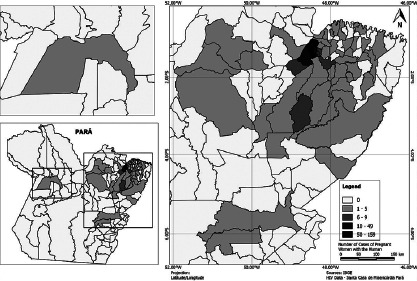
Geographical distribution of Human Immunodeficiency Virus cases in pregnant women hospitalized in a public referral Maternity Hospital in the state of Pará from 2010 to 2019, according to municipality of origin, Belém, Pará, Brazil, 2020

The thematic map was generated based on the identification of the cities of residence of pregnant women living with HIV hospitalized in the period and the analysis performed. The classification adopted included five classes of intervals, allowing the spatial distribution of the number of cases of pregnant women who tested positive for HIV in the state of Pará; 66.87% belonged to the Metropolitan Region, including municipalities of Ananindeua, Marituba, Santa Izabel do Pará, Benevides, Castanhal and Santa Barbara do Pará, and 33.13% belonged to the inland of the State (Figure 1).

In multivariate analysis, variance was given by components that expressed eigenvalues, with the first components being the main responsible for explaining the variance of data, since eigenvalues were directly proportional to the principal components. That is, the highest eigenvalue was associated with the first principal component and the lowest with the last principal component.

**Figure 2:**
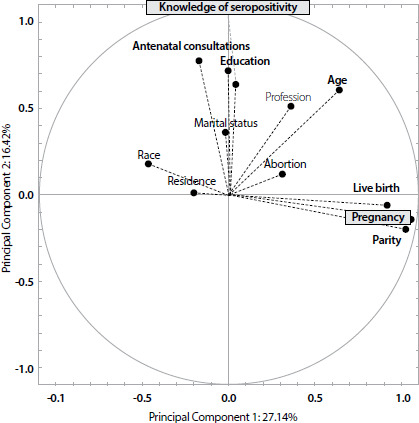
Multivariate circle of correlation between variables associated with the prevalence of Human Immunodeficiency Virus infection in pregnant women hospitalized from 2010 to 2019, Belém, Pará, Brazil, 2020

Thus, pregnancies, number of live children and deliveries were the variables with higher eigenvalue in the first component. In the second component, the variables with higher values were education, age, race, marital status, profession/occupation, municipality of residence, number of abortions, knowledge of serological status and number of antenatal consultations. Thus, these variables had the greater association and influenced the variance of data, as demonstrated by the multivariate circle representing the behavior of variables. Those closer to the edges of the circle are the most important for data variance and those grouped by proximity have greater correlation (Figure 2).

By understanding that the closer to value 1.0, the greater the influence on data variance, the variables pregnancies (0.96), number of live children (0.83), deliveries (0.93), education (0.65), knowledge of serological status (0.58) and number of antenatal consultations (0.70) were the most important. As for correlation, the education variable had a greater correlation with the number of antenatal consultations, and marital status had a greater correlation with knowledge of serological status.

## DISCUSSION

The results described showed an increase in the rate of infection in the period studied and the concentration of cases in the Metropolitan region, with a significant number of pregnant women living with HIV having their diagnosis confirmed only during their admission to the maternity ward, that is, they were unaware of their serological status and, consequently, did not undergo ART, increasing the risk of VT. There was a strong relationship between the number of antenatal consultations and lack of knowledge of serological status variables, and a correlation between education and the number of antenatal consultations variables, and between marital status and knowledge of serological status variables.

The increase in number of HIV cases in this population confirms the need to redefine programmatic actions in the state and recognize the constraints of female vulnerability in the context of HIV/AIDS^(12)^. Infection is associated with adverse pregnancy outcomes and all pregnant women living with HIV, regardless of their clinical stage, should receive a combination of antiretroviral drugs to suppress maternal viral load and prevent VT^(13)^.

The analysis of the sociodemographic profile of the sample showed characteristics similar to other studies that evaluated women with HIV^(14,15,16)^; predominance of the age group 20-30 years, mixed race, in a common-law marriage, complete regular education and housewives. Given the feminization of the HIV epidemic, socioeconomic factors can act as determinants for the infection, since most infected women were still of childbearing age^(14)^, in a common-law marriage^(17)^, mixed race^(18)^, housewives^(19)^ and have a low level of education.

There was a predominance of multiparous pregnant women who underwent antenatal care, although an expressive number had not had the ideal number of six antenatal consultations recommended by the Ministry of Health^(20)^. This result is consistent with a study that used data from the “*Nascer no Brasil*” (Being born in Brazil, in English) study and databases of the National Information Systems, in which although 95% of participants received antenatal care, only 29.4% had the recommended minimum number of six consultations^(3)^.

Antenatal Care is a set of actions that are simultaneously preventive, health-promoting, diagnostic and curative, aiming at the good outcome of the pregnancy for the woman and her child(ren)^(21)^. In the context of HIV identification, pregnant women who receive inappropriate antenatal care may be less likely to adhere to appropriate therapy and achieve viral suppression at delivery. Certainly, adherence to antenatal consultations becomes an important motivator for women achieving good health outcomes for them and their babies, facilitating repeated assessments of the infection^(22)^.

This may explain why, in this study, of 46.99% pregnant women diagnosed with HIV, 15.66% were unaware of their serologi-cal status and 25.90% did not undergo ART. This finding was reinforced by bivariate analysis, in which a strong relationship between the number of antenatal consultations and lack of knowledge of serological status (p value equal to 0.01^E-17^) variables was found. These results may demonstrate the delay in finding these pregnant women, either because of their lack of interest in adherence, the difficulty in accessing health services or even the difficulty of services themselves in guiding and approaching these pregnant women, highlighting the importance of early diagnosis and adherence to prophylactic measures since antenatal care^(14)^.

In Brazil, due to failures in the early diagnosis of HIV during antenatal care, as identified in this study, since 2015, all pregnant women hospitalized for childbirth must undergo the rapid test, regardless of the date of the last test, taking into account the false negatives within the immunological window, a fact that helped in a more reliable diagnosis among pregnant women and in the homogenization of protocols in maternity hospitals at the national level^(20)^. Routine HIV screening has been conducted in various clinical settings using different testing methodologies, including point-of-care rapid testing, processes for obtaining patient consent, and strategies for reporting results^(23)^.

Therefore, monitoring these tests can help to visualize the prevalence of infection in pregnant women. In the present study, 20.69% of positive tests in the period were performed at the Urgency and Emergency Department and there was an increase in HIV prevalence of pregnant women hospitalized at the Institution, since in 2010, the prevalence was 2.72% and in 2019, it reached 2.83%.

This increase becomes more significant when comparing these results with the last prevalence study conducted at the Institution in 2011, in which the medical records of 770 pregnant women treated between 2004 and 2010 were analyzed and the HIV prevalence rate of 1.87% was found^(6)^. In our study, the infection prevalence rate in the studied period was 2.39%, indicating a significant increase. These data are corroborated by a study conducted with HIV cases in pregnant women residing in the state of Pará reported to the Notifiable Diseases Information System (Portuguese acronym: SINAN) in the period from 2010 to 2017. A trend of increase in the detection rate of HIV in pregnant women was identified in recent years, with an average annual growth of 0.8%^(12)^.

A cross-sectional study^(24)^ conducted in a Chinese province over seven years found an increase in the prevalence of HIV in pregnant women, ranging from 0.75% to 6.6% in regions of the area in 2015, considering that in 2002 , other on-site surveys found no HIV positive cases. To deal with the disproportionate burdens of HIV infection in pregnant women, which also serve as a barrier to the elimination of new pediatric infections, it is imperative to understand the clinical, social and geographical factors associated with this high burden of HIV^(7)^.

In this context, in the assessment of the distribution of pregnant women living with HIV, most were from the Metropolitan region. In a study conducted in Belém, PA, with pregnant women living with HIV in follow-up treatment at the Specialized Care Service of a University Hospital, the majority also (82.97%) came from the Metropolitan region, mainly from the capital Belém^(25)^. This result differs from a study conducted in southwest China^(26)^, in which 92% of pregnant women living with HIV came from rural areas hence, women in unfavorable economic and social conditions with more limited education were more affected than those living in urbanized areas.

The urbanization and development in big cities are known to influence the spread of infections, especially HIV^(27)^. A study conducted in Catalonia, Spain, identified that the highest rates of new HIV diagnoses occurred in regions located in urban areas, especially in the city of Barcelona^(28)^. Another study conducted in the United States revealed statistically significant differences in the distribution of new diagnoses by demographic characteristics and urban environment, with higher HIV positivity rates in urban areas^(29)^. These results corroborate the findings of the present study.

Another aspect underlying the high rates of contamination in these areas are the various behaviors commonly adopted by individuals living in urban locations, such as not using condoms, early age at sexual initiation and multiple sexual partners. In addition, greater sexual freedom among young people is related to the increasingly early occurrence of first sexual intercourse among Brazilian women^(30)^.

These results may be associated with the fact that rapid urbanization and population growth can generate social inequalities, such as low income, inadequate housing, poverty and difficulties in accessing education and health systems, which favor the spread of HIV^(31)^. From this understanding, it is possible to infer that such inequalities generate a strong impact on awareness of the disease and the methods of prevention.

A cross-sectional study performed in ten regions of Cameroon, Africa, showed an association between educational level and HIV infection in pregnant women in the bivariate analysis, although the educational level was not significant in the multivariate analysis^(7)^. This finding differs from the present study, because in the multivariate analysis, the education variable had a strong influence on data variance and a greater correlation with the number of antenatal consultations.

The low schooling of pregnant women is highlighted as a possible limiting factor of the full implementation of preventive measures, since women with less schooling commonly depend on public health services and are less likely to start antenatal care at the beginning of pregnancy or to complete the recommended number of antenatal consultations^(32)^.

A study conducted in Zambia, Africa, showed a strong association between educational level and HIV testing when adjusted for variables such as age, wealth index and location, which are similar results to the analysis of the present study. This means that sociodemographic factors, including the woman’s level of education and health system factors, can influence the accessibility and adherence of these pregnant women to HIV and VT prevention services^(33)^.

As for the correlation of the marital status variable with knowledge of serological status, this may reflect the historical construction and understanding of female roles in marital relationships. In the context of HIV/AIDS, most women living with the infection are in a steady relationship, usually married or in a common-law marriage, living with their partner. However, the dimensions of the type of sexual partnership are not well listed in the literature as an individual factor for HIV transmission and on the other hand, being in a steady relationship favors the inconsistent use of condoms and the consequent increase in vulnerability to HIV, as well as the early diagnosis and treatment^(34)^.

Thus, there is the assumption that pregnant women have less time for self-care such as prophylactic measures and antenatal care, which associates marital status with the risk of mother-to-child transmission of HIV^(35)^, and reinforces that health should not only be directed to the availability of health services, but also to self-care capacity, influenced by the educational level and marital status of the pregnant woman^(36)^.

A South African study on HIV and sociodemographic and behavioral determinants^(37)^ showed that married individuals and/or living with a spouse had significantly less chances of being infected with HIV compared to single people, indicating an association between marital status and the prevalence and incidence of HIV in the country, so HIV prevention needed to be better targeted at single populations. This result is opposite to that found in the present study, since there was a predominance of populations in a common-law marriage.

However, the study also found that some risk factors for HIV are reportedly higher for married women and those in a common-law marriage, as they are less likely to use condoms compared to single women. Furthermore, married women and those in a common-law marriage were less likely to discuss HIV with their partners, suggest condom use, and were more likely to experience higher rates of male infidelity in their relationships than single women, which could lead to high levels of infection^(37)^.

The epidemiological profile of people infected with HIV/AIDS has undergone several changes over the years. At first, the most affected population group consisted of male homosexuals and bisexuals, people who underwent blood transfusions and injecting drug users. Currently, the spread of infection has grown considerably among the female population, given the phenomenon of heterosexualization and feminization of the disease, a situation that has a strong impact on public health and the increase in the number of pregnant women living with HIV^(38)^.

Therefore, data collection clearly demonstrates the importance of offering specialized services for these infected women considering their socioeconomic profile; mostly mixed race with low educational level, who need to be assisted and oriented regarding contraception and prevention of sexually transmitted infections^(18)^.

Considering the pregnancy-puerperal scope, it is also necessary to implement actions aimed at expanding care to women, ensuring comprehensive care in line with early diagnosis as an important strategy to promote appropriate adherence to treatment and reduction of VT, placing health professionals as main protagonists in clinical decision-making, with provision of important information for the primary health care of pregnant women in the pregnancy cycle. In this context, nurses play a fundamental role as educators/facilitators committed to using health education strategies in an attempt to encourage changes in risky behavior and family planning^(18)^.

In addition to pre- and post-test counseling, nurses’ role in preconception counseling also stands out, when they inform women living with HIV about the risk of VT, the means available to avoid it, and accessible contraceptive methods, thereby giving women the possibility to choose or not a future pregnancy. Health professionals must be available for dialogue and engaged with issues of gender, sexuality and reproductive health without losing sight of the ethical, social and cultural dimensions that regulate the lives of women living with HIV, discussing their decisions and desires in care, aiming to provide safer recommendations for family planning, and information on the care needed during pregnancy, childbirth and puerperal period, in addition to respect for their rights as citizens^(39)^.

### Study limitations

The limitations of this study are related to the fact that secondary data were used, as there may be problems with the filling out and incomplete information, leading to failures in investigations. In addition, the fact that this was a convenience sample performed in a referral Maternity Hospital in the state of Pará makes it difficult to generalize the findings that only represent patients from the institution where the study was developed.

### Contributions to the area of Nursing, Health or Public Policy

The findings of this study allowed the survey of the HIV prevalence and associated factors in pregnant women in the state of Pará, as well as the recognition of vulnerabilities in this public. This information can support the planning of measures and strategies for the prevention and control of this condition, ensuring the quality of care and contributing to the reflection of health professionals, especially nurses, who need to be aware of biological, psychological, clinical and social demands of pregnant women living with HIV. The nurse has a prominent role in antenatal care, ensuring the qualification of health services and early detection of diseases that may affect these women in the pregnancy cycle through actions that guarantee the performance of rapid tests, favoring the reduction of HIV prevalence in pregnant women in the state.

## CONCLUSIONS

From the results of the study, the prevalence of HIV and the associated factors in pregnant women in the state of Pará could be analyzed, noting an increase in the rate of infection in pregnant women in situations of vulnerability in the period studied. This demonstrated the need to intensify health actions, early diagnosis and strategies to improve adherence to anti-retroviral treatment for maternal viral suppression and reduce the risk of vertical transmission, contributing to improve public policies in the state.

When finding the strong relationship between the lack of knowledge of serological status and schooling with antenatal care, the need to implement actions aimed at expanding care for pregnant women became evident, especially antenatal care, ensuring comprehensive care and early diagnosis, which are strategies necessary for the promotion of adequate adherence to treatment and reduction of VT, with nursing professionals playing a protagonist role.

These findings are expected to provide support for further studies, thus strengthening discussions on care aimed at pregnant women and HIV prevention at state, regional and national levels, favoring the development of strategic actions that consider the peculiarities and specific needs of pregnant women.
